# The genome-wide landscape of C:G > T:A polymorphism at the CpG contexts in the human population

**DOI:** 10.1186/s12864-020-6674-1

**Published:** 2020-03-30

**Authors:** Jeonghwan Youk, Yohan An, Seongyeol Park, June-Koo Lee, Young Seok Ju

**Affiliations:** 1 Graduate School of Medical Science and Engineering, Korea Advanced Institute of Science and Technology (KAIST), 291 Daehak-ro, Yuseong-gu, Daejeon, 34141 Republic of Korea; 2 Biomedical Science and Engineering Interdisciplinary Program, Korea Advanced Institute of Science and Technology (KAIST), 291 Daehak-ro, Yuseong-gu, Daejeon, 34141 Republic of Korea

**Keywords:** CpG, CpG island, Single nucleotide polymorphism, Transition, Methylation

## Abstract

**Background:**

The C:G > T:A substitution at the CpG dinucleotide contexts is the most frequent substitution type in genome evolution. The mutational process is obviously ongoing in the human germline; however, its impact on common and rare genomic polymorphisms has not been comprehensively investigated yet. Here we observed the landscape and dynamics of C:G > T:A substitutions from population-scale human genome sequencing datasets including ~ 4300 whole-genomes from the 1000 Genomes and the pan-cancer analysis of whole genomes (PCAWG) Project and ~ 60,000 whole-exomes from the Exome Aggregation Consortium (ExAC) database.

**Results:**

Of the 28,084,558 CpG sites in the human reference genome, 26.0% show C:G > T:A substitution in the dataset. Remarkably, CpGs in CpG islands (CGIs) have a much lower frequency of such mutations (5.6%). Interestingly, the mutation frequency of CGIs is not uniform with a significantly higher C:G > T:A substitution rate for intragenic CGIs compared to other types. For non-CGI CpGs, the mutation rate was positively correlated with the distance from the nearest CGI up to 2 kb. Finally, we found the impact of negative selection for coding CpG mutations resulting in amino acid change.

**Conclusions:**

This study provides the first unbiased rate of C:G > T:A substitution at the CpG dinucleotide contexts, using population-scale human genome sequencing data. Our findings provide insights into the dynamics of the mutation acquisition in the human genome.

## Background

Cytosines at the CpG dinucleotide sequence contexts are frequently methylated in vertebrate genomes [1, 2]. The 5-methyl cytosines are susceptible to spontaneous deamination to thymine [3]. If DNA repair mechanisms fail to remove the mutated T with a G on the opposite strand before DNA replication [4–6], C > T substitutions (referred to by the pyrimidine of the mutated Watson-Crick base pair) are fixed in one of the two daughter cells and maintained in the descendant cells. In the vertebrate genome, in which 5-methyl cytosines are enriched at the NpCpG sequence context (where *N* = A, C, G or T). As a result, the rate of C > T transition at the CpG sequence contexts is ~ 12 times higher than other transition types [7]. As a result, 5-methyl cytosine deamination is one of the major source of de novo mutations and somatic mutations occurred even in the early embryogenesis, constituting approximately 20–30% of novel DNA polymorphisms [8–10]. In theory, these mutations can be permanently fixed, present as polymorphic site, or completely lost in the human population, depending on selection and genetic drift. Because of the relatively high mutation rate, CpG sequence contexts have been depleted over time, being present approximately 25% compared to what is expected from the nucleotide composition of the reference human genome sequence [11].

The genome-wide distribution of CpG is not random [12], suggesting the mutation rate in each CpG varies according to the genomic location and/or to the nature of each CpG [13–15]. For example, CpG islands (CGIs), the genomic regions where CpGs are exceptionally enriched [16], are generally known to be resistant to C > T substitution because cytosines in CGIs are mostly hypo- or unmethylated unlike non-CGI CpGs [17]. In addition, all CGIs are not homogeneous in regards to CGI lengths, functional context in the genome and methylation levels [16, 17]. For example, approximately half of all CGIs contain transcription start sites (TSSs) for the genes, and the others are distant from the TSSs [18]. Interestingly, despite the high mutation rate at CpGs and > 100,000 years of human evolution, many non-CGI CpGs are still present in the human genome sequence. Presumably, active mutational processes and/or purifying selection processes are ongoing for these cytosines in the pool of human genomes. Although the landscape CpG mutations have been previously reported in the human genome [14, 15, 19], the limited sample size and uneven coverage for genome-wide CpGs have not enabled a comprehensive analysis of the mutation dynamics of CpGs in the human population. Recently, a map of human genome variations has been made from more than 4300 whole-genome sequencings of normal individuals from the 1000 Genomes Project Consortium [20–22] and the Pan-Cancer Analysis of Whole Genomes (PCAWG) projects of International Cancer Genome Consortium (ICGC) [23, 24]. Moreover, protein-coding genetic variations have been more deeply investigated from more than 60,000 whole exome sequencing by the Exome Aggregation Consortium (ExAC) [25]. Using these large databases, we investigate the landscape and dynamics of germline mutations at the CpG contexts in the human genome.

## Results

### C > T substitutions in CGIs

**Fig. 1:**
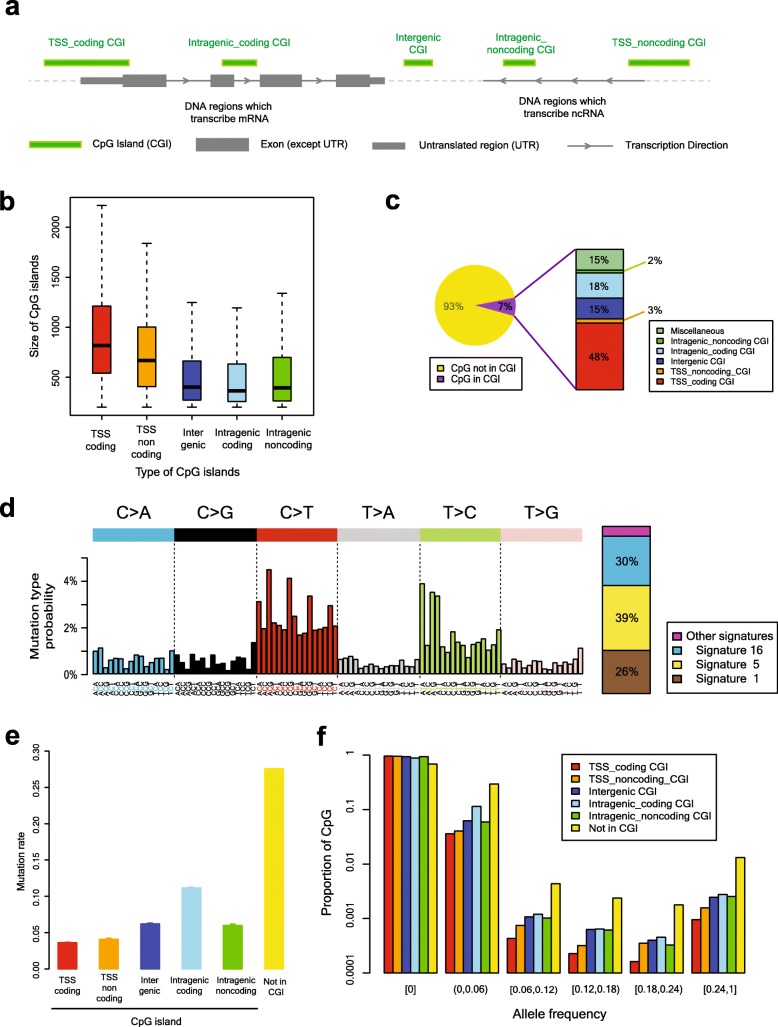
C > T polymorphism rate in the human population and classification of CpG islands (CGIs). **a** Schematic illustration of the classification of CGIs. **b** The distribution of the size of the CGIs according to the CGI types. CGIs related to a transcriptional start site (TSS) are significantly longer compared to the others. **c** The statistics of CpG dinucleotides in the reference human genome. Among C or G at the CpG dinucleotide sequence context, approximately 7% are located in CGIs. Approximately half of the CpGs in CGIs are located in the TSS-coding CGIs. **d** Mutational spectrum accumulated during human genome evolution. Decomposition of the mutational spectrum revealed that C > T transitions at the CpG contexts (Signature 1) were one of three major signatures during human genome evolution. **e** Mutation rate of CpGs based on CGI classifications (Error bars indicate 95% confidence intervals). Interestingly, intragenic coding CGIs have the highest mutation rate among the five CGI types. **f** The distribution of allele frequencies of the C > T transitions according to the CGI types. As the higher the mutation rate of the CpGs in **e** becomes higher, the absolute value of allele frequencies tends to be higher. A logarithmic scale is applied to the y-axis

Human reference genome sequence (GRCh37) harbors 28,084,558 CpGs. First, we investigated the landscape of the C > T substitutions at the CpGs in all 27,718 CGIs in the human genome. We categorized those CGIs into five types according to functional contexts (Fig. 1a; Methods). These CGI types showed different size distributions (Fig. 1b): TSS containing CGI types were significantly longer than other types (*p*-value < 0.001; Kruskal-Wallis test). About 7% (2,073,739) of the CpGs were located in CGIs (Fig. 1c, Additional file 1: Table S1). Of these CGI CpGs, approximately half of the bases (48%) were located in TSS-coding CGIs, followed by intragenic-coding CGIs (18%), intergenic CGIs (15%), TSS-noncoding CGIs (3%), and intragenic-noncoding CGIs (2%).

Using 4327 whole genome sequences from the 1000 Genomes [20] and the PCAWG [23, 24] projects, we found 14,579,739 C:G > T:A mutation events from all CpGs (26.0%). We estimated that its underlying mutational process (termed Signature 1) [26] accounted for 26% of the mutations recently acquired in the human gene pool (Fig. 1d; Methods).

Consistent with conventional knowledge, the mutation rate of the CpGs in CGIs was much lower than that of the non-CGI CpGs (5.6 and 27.6%, respectively; *p* < 0.001; chi-squared test). Interestingly, each CGI type had a dissimilar C > T mutation rate. Especially, the C > T substitution rate in the intragenic-coding CGIs was substantially higher than that of the TSS-coding CGIs, TSS-noncoding CGIs, intergenic CGIs, and intragenic noncoding CGIs (Fig. 1e, Additional file 1: Table S1; 11.2% versus 3.6, 4.1, 6.2, and 6.0%, respectively; *p* < 0.001; chi-squared test). This tendency of different C > T substitution rate depending on the CGI types was consistently observed in each sequencing project (Fig. S1a), in each ethnicity (Fig. S1b), and even in individual data (Fig. S1c).

To consider the population minor allele frequency of the C > T variants, we calculated the C > T substitution rate of each CGI type for six ranges of allele frequencies (Afs) as follows: Af = 0, 0 < Af < 0.06, 0.06 ≤ Af < 0.12, 0.12 ≤ Af < 0.18, 0.18 ≤ Af < 0.24, and 0.24 ≤ Af. The substitution rate at the CpG contexts in the intragenic coding CGIs was the highest for all ranges of Af > 0 (Fig. 1f, Additional File 1: Table S2; *p* < 0.001 for each interval of positive allele frequencies; chi-squared test). In addition, we also calculated the Af-weighted C > T substitution rate (Methods), and the tendency of the Af-weighted rate was consistent with the above results (Fig. S2).

The CpGs in the TSS-coding CGIs and the intragenic-coding CGIs and the CpGs not in the CGIs were located at either protein-coding exons or non-coding regions. Given that the two genomic contexts could be under different selective pressures, we divided each CGI type into protein-coding exons and non-coding regions, and analyzed the C > T substitution rate. Interestingly, the mutation rate of each region was more affected by the CGI types rather than by the protein-coding exons or not (Fig. S3a). In addition, the protein-coding exons in the TSS-coding CGIs and the intragenic-coding CGIs had higher mutation rates than those of the non-coding regions (*p* < 0.001; chi-squared test), which were in contrast to the trend at the CpGs not in the CGIs. Because it was observed that negative selection pressure in the noncoding regions of the two CGI types was higher than that of the non-CGIs, we thought that the non-coding regions in the TSS and gene body might have an important role in human reproduction.

### C > T substitutions in CGI shores

**Fig. 2:**
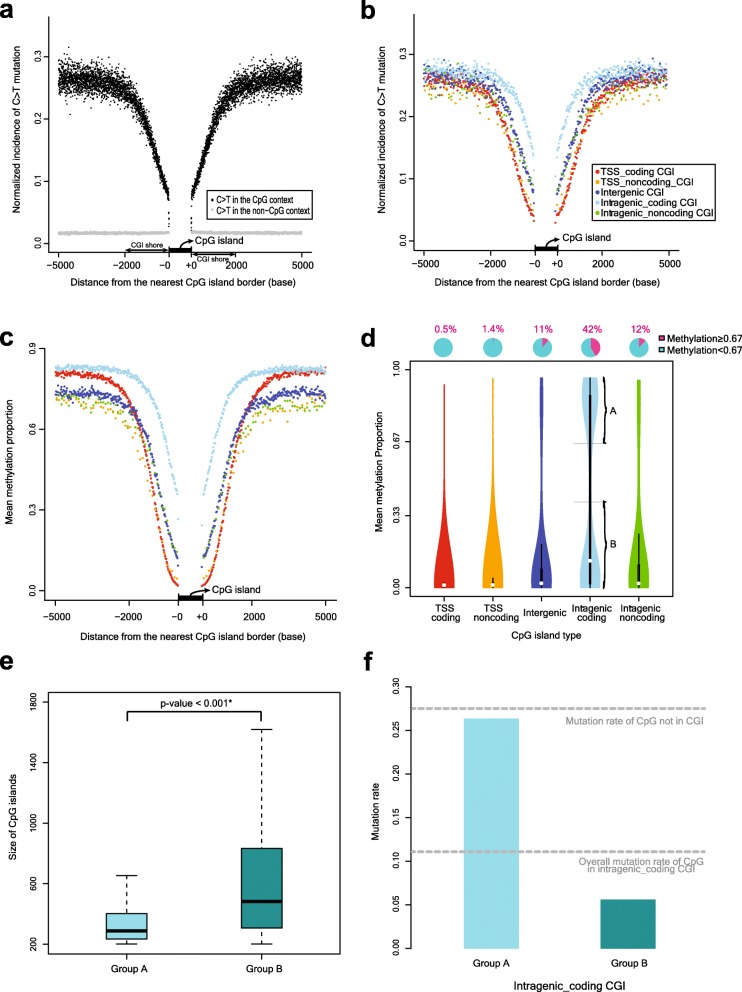
C > T polymorphism rate and methylation proportion around each type of CGIs. **a** Normalized incidence of C > T mutation according to the distance from the border of the CGIs. In the CGI shores, as the distance from the border of CGI becomes closer, the mutation rate of C > T is lower. Beyond 2 kb, the mutation rate of C:G > T:A plateaus. As a control, the normalized incidence of the C > T mutation at the non-CpG contexts is also depicted as grey dots. **b** The normalized incidence of the C > T mutation for each CGI type. In the CGI shores of the intragenic-coding CGIs, the incidence of C > T mutations is higher compared to the other CGIs. The normalized incidence of C > T mutations in the TSS-coding CGIs and TSS-noncoding CGIs tended to be lower than that of the non-TSS CGIs. **c** The pattern of the mean methylation percentage in each CGI type according to the distance from the border of CGIs. On the whole, the methylation percentage in the CGI shores is well correlated with the order of the normalized incidence of C > T mutations shown in (B). **d** Violin plots of the distribution of the mean methylation proportion according to each CGI type. The methylation pattern of the Intragenic-coding CGIs uniquely shows the bimodal distribution. **e** Intragenic coding CGIs with a mean methylation of > 67 and < 33% are classified as group A and B, respectively. Interestingly, the size of the CGIs in Group A is significantly shorter than that of the CGIs in Group B. **f** Mean mutation rate of the CGIs in Group A and B

Next, we assessed the CpG mutation dynamics in the vicinity of CGIs. The normalized C > T substitution rate at the CpGs increased to 2 kb (CGI shores) from the nearest boundary of the CGIs (Fig. 2a). Beyond 2 kb, the mutation rate became constant. The increasing-and-plateau pattern was observed for the C > T substitutions only in the CpG contexts and not in other sequence contexts such as CpA, CpC and CpT (grey dots in Fig. 2a). The normalized rates of the C > T substitution in the non-CpG contexts were much lower (less than 0.025) than those in the CpG contexts. Because the mutation rate of the CGI CpGs was different among the CGI types (Fig. 1e and f), we compared the C > T mutation patterns for the shores of the different CGI types. Because the mutation rate of C > T in the intragenic-coding CGIs was the highest among the five CGI types, the normalized incidence of the C > T mutation in the CGI shores was also the highest around the intragenic-coding CGIs (Fig. 2b; *p* < 0.001; paired t-test). The CGI shores in the intergenic CGIs and intragenic-noncoding CGIs were the next, followed by the TSS-coding CGIs and TSS-noncoding CGIs. Beyond 2 kb from the CGI borders, the normalized incidences of the C > T mutations were similar among the five CGI types.

To understand the underlying mechanism of the difference in the C > T mutation frequency among the different types of CGIs, we correlated the mutation rate with the average methylation level (beta values) of cytosines in the CGIs and CGI shores (Fig. 2c). Consistent with the substitution rate, the mean methylation level was the highest in the intragenic-coding CGIs (*p* < 0.001; paired t-test), starting from approximately 0.3 at the border to approximately 0.8 at the ±2000 bp position. Similar to the order of the C > T mutation incidence, the intergenic CGIs and intragenic-noncoding CGIs followed next in terms of the mean methylation level. The mean methylation proportions of the CGI shores in the TSS-coding CGIs and TSS-noncoding CGIs were the least among the five CGI types. The non-CGI CpGs outside of the CGI shores had high methylation levels between 0.6 and 0.9. Interestingly, the CpGs inside the CGIs had different methylation levels according to the CGI types. The mean methylation levels of each CGI type were 0.018, 0.039, 0.139, 0.411, and 0.154 for the TSS-coding CGIs, TSS-noncoding CGIs, intergenic CGIs, intragenic-coding CGIs, and intragenic-noncoding CGIs, respectively. This methylation pattern was well correlated with the mutation rate for each CGI type (Fig. 1e).

At face values, the intragenic-coding CGIs had significantly higher mean substitution and mean methylation levels compared to the other CGI types. However, we further realized that the intragenic-coding CGIs were remarkably sub-classified into two heterogeneous groups, hyper-mutated/hyper-methylated and hypo-mutated/hypo-methylated groups (Fig. 2d). For example, approximately 40% of the intragenic coding CGIs were hyper-methylated (mean beta value > 67%; Group A) and the vast majority of the rest were hypo-methylated (mean beta value < 33%; Group B). The CGIs in the hyper-methylated group were much smaller in length (Fig. 2e; *p* < 0.001; Wilcoxon rank sum test) and had a higher C > T mutation rate (Fig. 2f) compared to those in the hypo-methylated group.

### Mutation rates according to the functional class of the C > T substitution in the protein-coding sequences

**Fig. 3:**
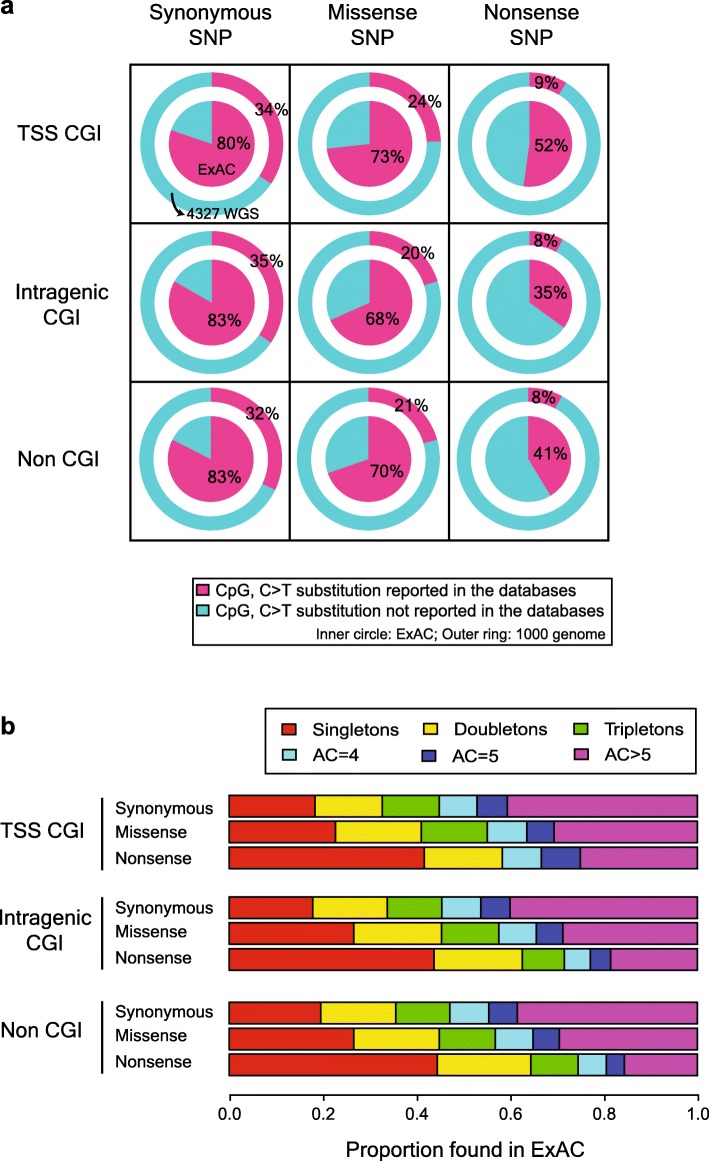
The difference in the C > T polymorphism rate according to the resulting amino acid changes. **a** Among the cytosines at the CpG dinucleotide sequence contexts in the coding sequences, the methylation of which is ≥67%, the proportions of reported C > T substitutions in the ExAC (inner circle) and the 1000 Genomes and the PCAWG (outer ring) database are illustrated. Nonsense-primed C > T mutations are negatively selected compared to missense and synonymous substitutions. **b** The distribution of the allele counts of C > T substitutions at the CpG contexts in the coding sequences in the ExAC database. Nonsense-primed C > T genomic loci have a singleton of more than 40%. As the effect of the amino acid change becomes smaller, the more the number of humans who have a C > T substitution on a specific locus increases, wherever a certain cytosine or guanine is located

C > T substitutions at the CpG contexts in the protein-coding regions can directly result in a change to the amino acid sequence of a protein (according to the genetic code context of a CpG) and in the phenotypes of an individual. Therefore, missense and nonsense mutations maybe under positive and/or negative selection. To assess the selective pressures, we explored C:G > T:A mutations at hyper-methylated CpGs (i.e. methylation levels ≥67%) in protein-coding sequences. For the analyses, we took advantage of a more extensive mutation catalogue from the ExAC Project (harboring protein-coding mutations obtained from > 60,000 individuals) and from the 1000 Genomes and the PCAWG projects (Fig. 3a, Additional file 1: Table S3). From 850 C or G at the CpG sites in TSS CGIs capable of silent mutations by C:G > T:A substitutions, we found 682 C:G > T:A substitutions (80%) from the ExAC dataset. Lower proportions of the C > T mutations were observed for the missense- and nonsense-primed sites (73 and 52%, respectively; *p* < 0.001; chi-squared test). In the intragenic CGIs and non-CGIs, silent mutation primed sites were more mutated compared to missense and nonsense mutation primed sites as well (Fig. 3a; *p* < 0.001; chi-squared test).

We further investigated the population minor allele frequencies of these C > T substitutions at the CpGs of the protein-coding regions (Fig. 3b, Additional file 1: Table S4). In line with our observation in the number of mutations (Fig. 3a), we found that the minor allele frequencies for missense and nonsense mutations were lower than those of the silent mutations. For example, the proportion of ultra-rare variants (singleton; allele count (AC) = 1) was the highest in the loci which would cause nonsense-primed CpGs by the C > T substitutions, followed by the loci for missense mutation and synonymous mutation. In contrast, the proportion of relatively common variants (AC > 5) showed an opposite trend.

Taken all together, missense and nonsense C > T mutations at CpGs in the TSS-CGIs, intragenic-CGIs, and at CpGs not in the CGIs were less frequently observed than expected for silent mutations in the human population, suggesting substantial levels of negative selection pressures for amino-acid changing and terminating mutations (Fig. 3a). This tendency was consistently observed regardless of our definition of hyper-methylation (Additional file 1: Table S5, Additional file 2: Fig. S4).

## Discussion

This study provided the dynamics of C > T substitution at CpGs in the human genome as a whole, in each type of CGIs, and in their vicinities including CGI shores, using uniformly covered whole genome sequencing data of 4327 people. The data reconfirmed the previous knowledge that CpGs in CGIs were generally more resistant to C > T transitions than non-CGI CpGs. In addition, CGI shores had a relatively lower mutation rate and lower mean methylation proportion compared to genomic regions other than CGIs.

The different features of the non-TSS CGIs (termed orphan CGIs) distinguished from the TSS CGIs were reported previously [17]. Of the three types of orphan CGIs (i.e., intergenic, intragenic-noncoding and intragenic-coding types), we found that a fraction of the intragenic coding CGIs have a substantially higher C > T mutation frequency presumably due to the hyper-methylated CpGs within. The features and functional consequences of the hyper-methylation of these intragenic coding CGIs have not been clearly understood [27]. In contrast to the general inhibitory function of methylation in the TSS CGIs, a few previous papers reported that hyper-methylation of gene body CGIs was observed in some genes with active transcription [28–30]. Despite their potential functional consequences, our findings suggest these intragenic-coding CGIs are being depleted more quickly in the human genome caused by spontaneous deamination of 5-methyl cytosines.

The recently published population-scale genome sequencing datasets enables us to assess the unbiased mutation rate at a genome-wide nucleotide resolution. Taking advantage of the results from the 1000 Genomes, the PCAWG, and the ExAC projects, we could observe the landscape of CpG mutations in the human population. Similar analyses using dataset obtained from several hundreds of thousands individuals will likely yield the dynamics of mutations at CpGs with a better resolution.

## Conclusions

This study has provided the genome-wide features of C > T mutations by spontaneous deamination in the human genome. It showed the continuous change of C > T transition rates around CGIs at the one base resolution. The mutation rate varies according to the context of the CGIs and to the selection pressures on the mutations. These findings could help to understand the rate and direction of genome sequence evolution in human populations.

## Methods

### Database sources

The human reference genome sequence (Build 37) was downloaded from the GATK website [31]. Sequences in conventional chromosomes, i.e. 22 autosomes and 2 sex-chromosomes, were analyzed in this study. In the analysis using allele frequencies, Y chromosome was excluded because the total number of alleles in each CpG locus of the Y chromosome was not consistent in the consensus call sets. Protein-coding genes and noncoding genes were annotated using RefSeq Gene, downloaded from the UCSC genome annotation database [32] on May 5th, 2016 [33, 34]. SNPs reported in the 1000 Genomes (phase 3), the PCAWG (ICGC; version 2.0), and the ExAC projects (release 0.3.1) were obtained from the UCSC genome annotation database [32], the PCAWG Germline Working group [24], and the ExAC Browser [35], respectively. In the case of the methylation analysis, whole-genome bisulfite sequencing data of human sperm was utilized because a substantial proportion of de novo C > T substitutions are believed to occur in the committed germline cellular lineages. The methylation data were downloaded from the MethBase [36, 37].

### Definition of CGI and its shores

We defined CGIs using the Gardiner-Garden’s criteria [16]: (1) GC content of 50% or greater, (2) length greater than 200 base pairs, (3) ratio greater than 0.6 for the observed number of CpG dinucleotides to the expected number based on the basis of the number of guanosines and cytosines in the segment. The positions of the defined CGIs were downloaded from the UCSC genome browser [32]. CGI shores were defined as the sequences within 2000 base pairs from the both sides of the CGIs [38].

### Classification of the CGIs

CGIs were further classified into intergenic CGIs, TSS-coding CGIs, TSS-noncoding CGIs, intragenic-coding CGIs, and intragenic-noncoding CGIs (Fig. 1a). If a CGI overlapped a TSS of a gene within ±100 base pairs, the CGI was designated as a TSS-coding CGI or a TSS-noncoding CGI, according to the types of genes (protein-coding or non-coding), respectively. The other CGIs that overlapped with genes were defined as intragenic-coding and intragenic-noncoding CGIs. If the CGI did not overlap with genes, the CGI was annotated as an intergenic CGI. The distance from the nearest CGI was assigned by the shortest distance from the border of the CGI. The distance was negative when a CGI was located in the upstream from the nearest CGI (and positive for a CGI located downstream of the nearest CGI). If a certain CGI belonged to more than one type of CGI, the CGI was classified as a miscellaneous CGI.

### Calculation of the mutation rate of the CpGs

The mutation rate of the CpGs in each CGI type was calculated as follows:

$$ mutation\ rate=\frac{the\ number\ of\ C:G>T:A\  mutated\ C\  or\ G\  at\  CpG\  contexts\ in\ each\  CGI\  type}{the\ number\ of\  all\ C\  or\ G\  at\  CpG\  contexts\ in\ each\  CGI\  type} $$

To consider the population allele frequencies (Afs) of the substitutions, the Af-weighted mutation rate was applied as follows:

$$ Af- weighted\ mutation\ rate=\frac{\varSigma\ Af\  of\ C:G>T:A\  mutated\ C\  or\ G\  at\  CpG\  contexts\ in\ each\  CGI\  type}{the\ number\ of\  all\ C\  or\ G\  at\  CpG\  contexts\ in\ each\  CGI\  type} $$

In the case of the mutation rate around the CGIs, the mutation rate of a certain distance (between − 5000 bp to + 5000 bp) from the close border of the nearest CGI was calculated as follows:

$$ mutation\ rate\ near\  CGI=\frac{the\ number\ of\ C:G>T:A\  mutated\ C\  or\ G\  at\  CpG\  contexts\  at\ a\  certain\ distance\ from\ the\ border\ of\ the\ nearest\  CGI}{the\ number\ of\ C\  or\ G\  at\  CpG\  contexts\  at\ a\  certain\ distance\ from\ the\ border\ of\ the\ nearest\  CGI} $$

When we depicted the mutation rate around each CGI, average values of 20 positions for the TSS-coding, intragenic coding, and intergenic CGIs and 100 positions for the TSS-noncoding and intragenic-noncoding CGIs were applied.

### Calculation of the mean methylation proportion of the CpGs.

The mean methylation proportion of the CpGs around each CGI type was calculated as shown below. When we showed the methylation proportion around each CGI, average values were applied as described in the above mutation rate section.

$$ mean\ methylation\ proportion\ near\  CGI=\frac{\varSigma\ \beta\  value\ of\ C\  or\ G\  at\  CpG\  contexts\  at\ a\  certain\ distance\ from\ the\ border\ of\ the\ nearest\  CGI}{the\ number\ of\  all\ C\  or\ G\  at\  CpG\  contexts\  at\ a\  certain\ distance\ from\ the\ border\ of\ the\ nearest\  CGI} $$

The mean methylation level in each CGI was calculated as follows:

$$ mean\ methylation\ level\ in\ each\  CGI=\frac{\varSigma\ \beta\  value\ of\ C\  or\ G\  at\  CpG\  contexts\ in\ each\  CGI}{the\ number\ of\  all\ C\  or\ G\  at\  CpG\  contexts\ in\ each\  CGI} $$

### Decomposition of the mutational spectrum

Thirty mutational signatures were previously generated using non-negative matrix factorization from more than > 10,000 whole exomes and whole genomes [39, 40]. To understand the mutational processes which have been mainly operative for de novo mutations during human evolution, we analyzed a mutational spectrum generated by substitutions rare in allele frequency (≤ 0.01 in the 1000 Genomes and the PCAWG projects; *N* = 103,068,067), because (1) rare SNPs were more likely to be acquired recently and (2) were much easier for assigning ancestral alleles. A multiple linear regression model was used to find the most proper combination of known mutational signatures, which were described previously [8]. Mutation signatures that explained less than 1% of the total rare SNPs were neglected.

### Statistics

Pearson’s chi-square test was used to investigate whether categorical variables differ from one another. Wilcoxon rank sum test was used to compare two groups of non-parametric continuous variables. Kruskal-Wallis test was applied in three or more groups of continuous variables. Dunn’s test was used in the post-hoc analysis. When we compared the mutation rate or mean methylation level in the vicinity of the intragenic-coding CGIs and other types of CGIs, paired t-test was applied using average values of every 20 position in each CGI between ±2000 bp from the border of the CGIs. All statistical calculations were performed with R version 3.1.3 (R Core Team, Vienna, Austria) [41]. A *p-value* of < 0.05 (two-tailed) was considered statistically significant.

## Supplementary information

**Additional file 1: Table S1.** Statistics of CpGs and CGIs in the human reference genome GRCh37. **Table S2.** The number of reported C > T polymorphism positions at the CpG dinucleotides according to their allele frequencies in the human population. **Table S3.** The number of reported C > T polymorphism positions at the CpG dinucleotides, methylation of which is ≥67%, in the protein coding sequences. **Table S4.** The number of reported C > T polymorphism positions at the CpG dinucleotides, methylation of which is ≥67%, in the protein coding sequences according to their allele frequencies in the human population. **Table S5.** The number of reported C > T polymorphism positions at all the CpG dinucleotides in the protein coding sequences.

**Additional file 2: Figure S1.** C > T substitution rate according to each CGI type in different populations. **Figure S2.** Af-weighted C > T substitution rate according to the CGI types. **Figure S3.** Af-weighted C > T. Fig. S3 C > T substitution rate in the protein-coding exons and non-coding regions in each CGI type. **Figure S4.** Proportions of the reported C > T substitutions at the all the CpG contexts in the coding sequences.

## Data Availability

The datasets analyzed during the current study are directly available in the UCSC genome annotation database [https://hgdownload.soe.ucsc.edu/gbdb/hg19/1000Genomes/phase3/], the ExAC Browser [https://storage.googleapis.com/gnomad-public/legacy/exac_browser/ExAC.r1.sites.vep.vcf.gz], and the ICGC Data Portal [https://dcc.icgc.org/api/v1/download?fn=/PCAWG/germline_variations/pcawg8.snps.indels.svs.phased.icgc.v2.controlled.vcf.gz]. Login is required to download the file from the ICGC Data Portal [https://dcc.icgc.org/releases/PCAWG/germline_variations/].

## Abbreviations

AC: Allele count
Af: Allele frequency
CGI: CpG island
ExAC: Exome Aggregation Consortium
ICGC: International Cancer Genome Consortium
PCAWG: Pan-cancer analysis of whole genomes
TSS: Transcription start site
