# Supplementary material for: The genome-wide landscape of C:G > T:A polymorphism at the CpG contexts in the human population
Source: BMC Genomics. 2020 Mar 30;21:270. doi: 10.1186/s12864-020-6674-1 (PMC7106825; doi:10.1186/s12864-020-6674-1)
Supplement: Supplementary file 2 — Additional file 2: Figure S1. C > T substitution rate according to each CGI type in different populations. Figure S2. Af-weighted C > T substitution rate according to the CGI types. Figure S3. Af-weighted C > T. Fig. S3 C > T substitution rate in the protein-coding exons and non-coding regions in each CGI type. Figure S4. Proportions of the reported C > T substitutions at the all the CpG contexts in the coding sequences. [file 12864_2020_6674_MOESM2_ESM.pdf]

Fig. S1

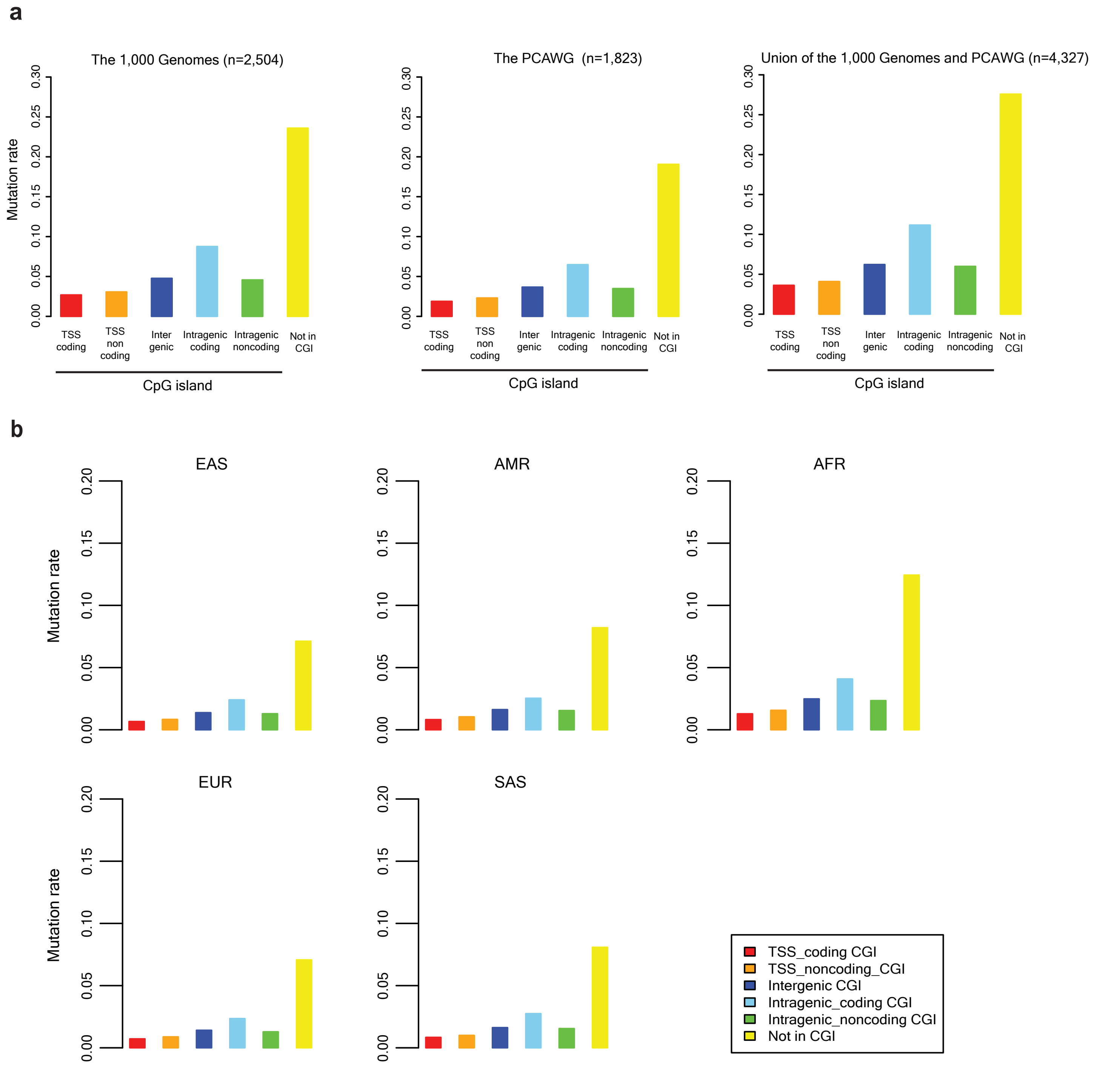

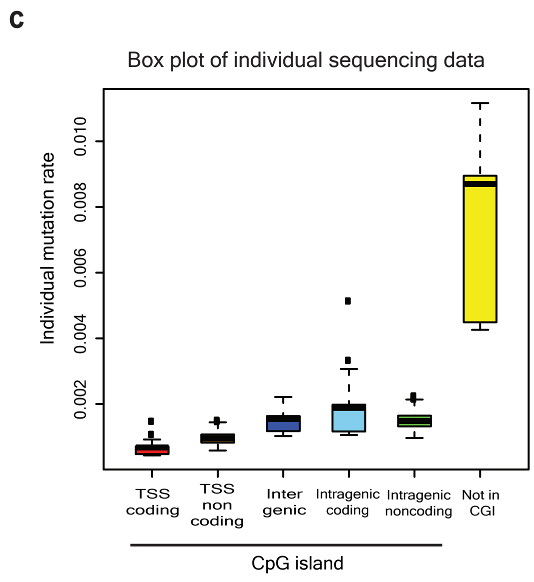

Fig S1. C > T substitution rate according to each CGI type in different populations. a In the 1,000 Genomes, PCAWG, and ExAC projects.
b The C>T substitution rate in each ethnic group from the 1,000 Genomes project including east Asian (EAS), American (AMR), African (AFR), European (EUR), and South Asian (ASA). **c** The C > T substitution rate from individual genotypes.

Fig. S2

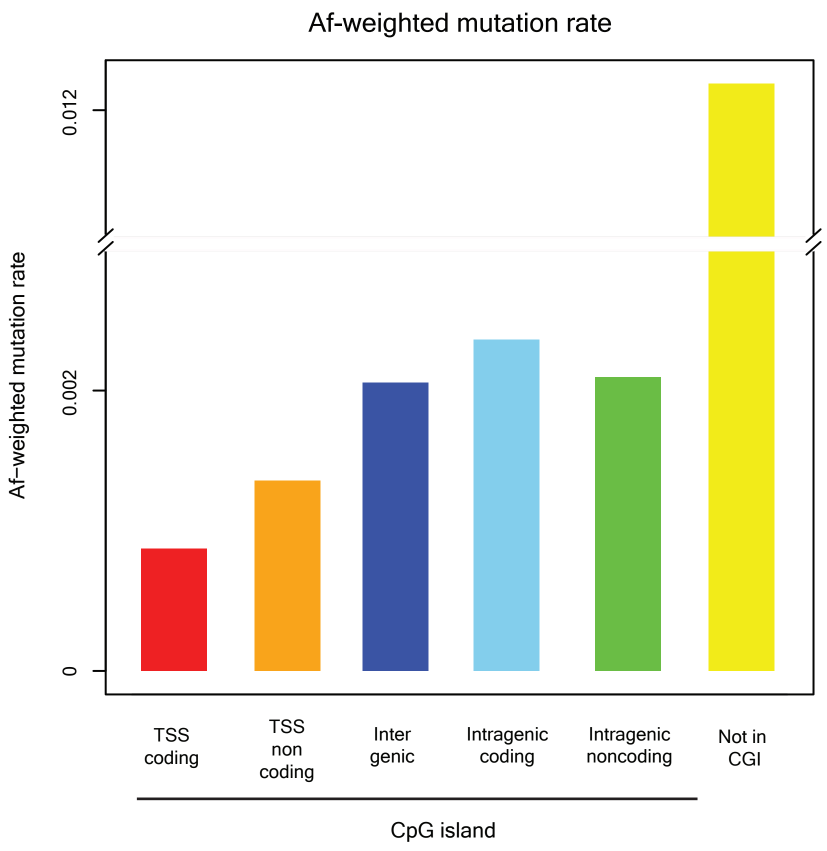

Fig S2. Af-weighted C>T substitution rate according to the CGI types.

Fig. S3

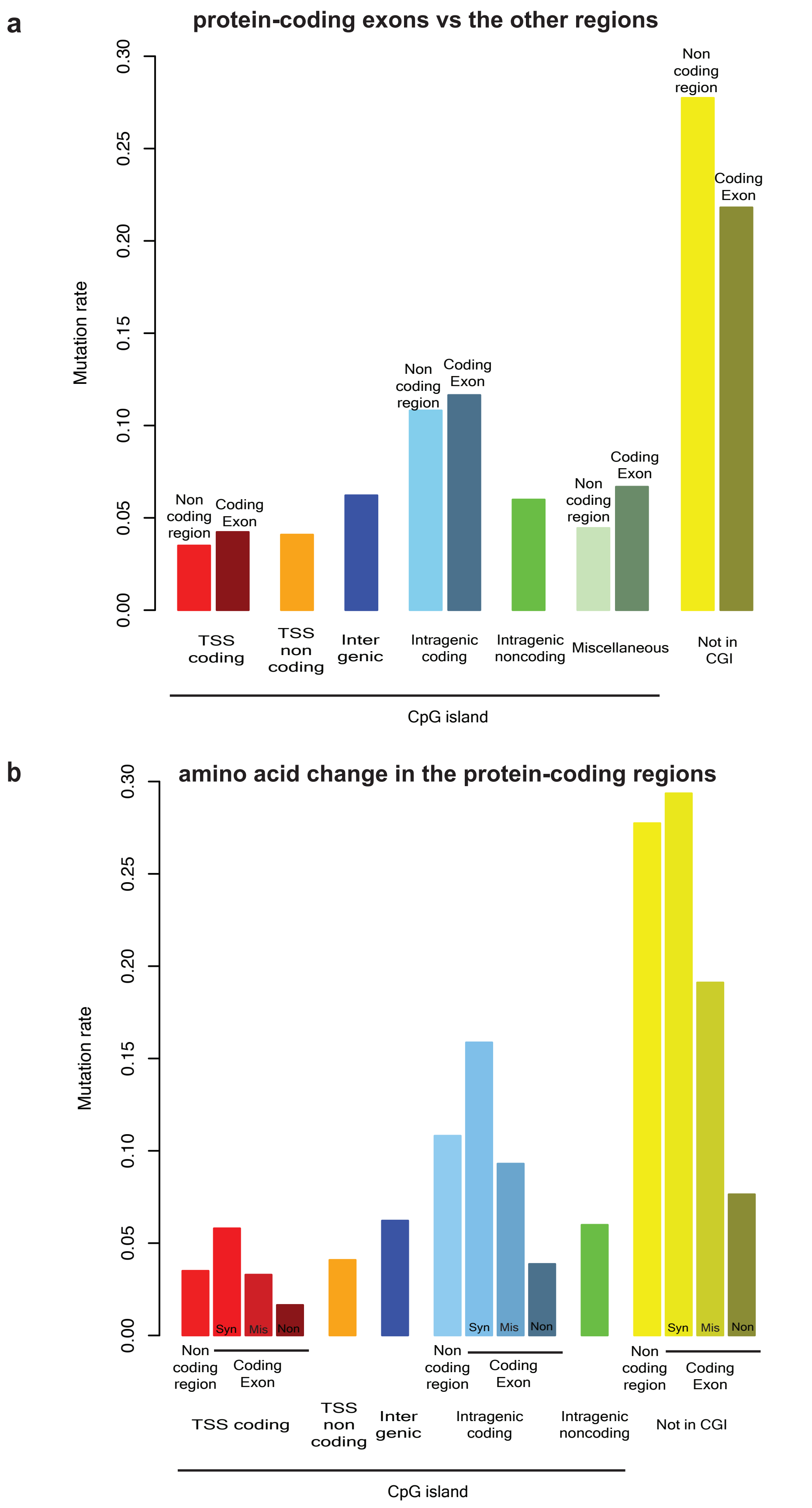

Fig S3. C > T substitution rate in the protein-coding exons and the non-coding regions in each CGI type. a C > T transition rate is more affected by the CGI types compared to whether certain CpGs are located in protein-coding exons or not. **b** The C > T transition rate in the non-coding regions has higher negative selection pressures compared to synonymous-primed loci. Interstingly, the non-coding regions in the TSS-coding and intragenic-coding CGIs have higher negative selecetion pressures compared to the non-CGIs. This implies that the non-coding sequences in a transcription start site or a gene body may be important in the human reproduction. Syn: synonymous-primed, Mis: missene-primed, Non: nonsense-primed

Fig. S4

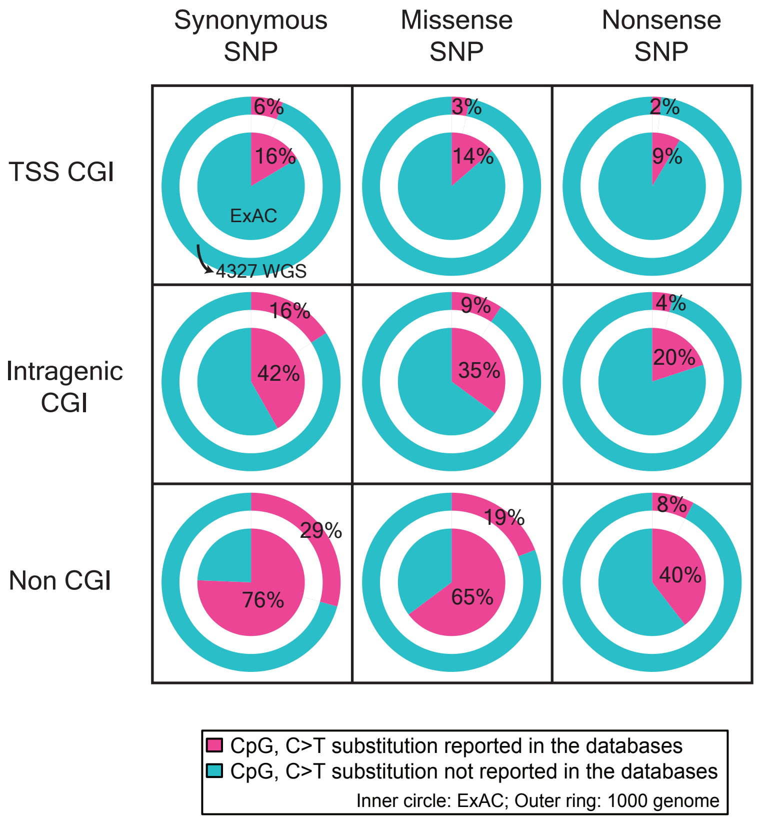

Fig S4. Proportions of the reported C > T substitutions at all the CpG contexts in the coding sequences. Among all cytosines (0% ≤ methylation level ≤ 100%) at the CpG dinucleotide sequence contexts in the coding sequences, proportions of the reported C > T substitutions in the ExAC (inner circle) and the 1,000 Genomes and PCAWG (outer ring) database are illustrated. Nonsense-primed CpG loci are negatively selected compared to missense-primed and silent-primed CpG loci.
